# Selective Lipoprotein Removal Enables High‐Purity EV Isolation from Plasma via Aptamer‐Based Mesh Filtration

**DOI:** 10.1002/smll.202514724

**Published:** 2026-03-03

**Authors:** SoYoung Jeon, YongWoo Kim, Sehyun Shin

**Affiliations:** ^1^ Department of Micro‐Nanosystem Technology Korea University Seoul Republic of Korea; ^2^ School of Mechanical Engineering Korea University Seoul Republic of Korea; ^3^ Engineering Research Center for Biofluid Biopsy Seoul Republic of Korea

**Keywords:** affinity, aptamer, high‐purity EVs, lipoprotein removal, mesh‐filtration, purification

## Abstract

Lipoproteins pose a major challenge to plasma extracellular vesicle (EV) isolation due to their abundance and physical similarity to EVs. Here, we present ApoFilter, a rapid and selective aptamer‐based mesh‐filtration platform that efficiently depletes ApoA1‐ and ApoB100‐positive lipoproteins. High‐affinity aptamers immobilized onto nylon mesh layers capture targeted lipoproteins in a spin‐column format, enabling rapid (1 min) separation under gravity‐driven flow. ApoFilter removed >99% of targeted lipoproteins (HDL and (V)LDL) from purified and plasma samples, with negligible capture of non‐target components and no compromise to EV integrity. When integrated with conventional EV isolation methods—including ultracentrifugation (UC), size‐exclusion chromatography (SEC), and ExoTFF—ApoFilter consistently eliminated lipoprotein contaminants across all workflows. This simple and rapid system addresses a key bottleneck in EV research, enabling the collection of ultrapure EVs suitable for downstream molecular analyses and translational applications.

## Graphical abstract

A novel affinity‐based mesh filtration system, ApoFilter, enables selective removal of plasma lipoproteins via sequential aptamer‐functionalized layers targeting ApoA1 and ApoB100. While EVs pass through unimpeded, (V)LDL and HDL are rapidly depleted. Compatible with various isolation workflows, this scalable platform yields high‐purity EV fractions for robust downstream molecular profiling and biomarker research.

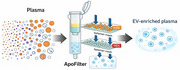

## 1 Introduction

Extracellular vesicles (EVs) are nanoscale lipid bilayer particles secreted by nearly all cell types and carry diverse biomolecular cargo—including proteins, lipids, mRNA, miRNA, and DNA—that reflects the physiological state of their cellular origin [1]. Their membrane encapsulation protects cargo in complex biological fluids and enables long‐range intercellular communication through targeted uptake and signaling modulation [2]. These unique properties have positioned EVs as promising agents for non‐invasive diagnostics [3] and therapeutic applications [4].

EVs are present in various biological fluids such as urine, saliva, cerebrospinal fluid, breast milk, and especially blood plasma, where they are found at concentrations as high as 10^9^–10^1^ ^2^ particles/mL. Plasma is therefore a rich reservoir for biomarker discovery; however, it is also one of the most challenging matrices for EV isolation. Plasma contains extremely abundant nanoscale lipoproteins, including HDL, LDL, and VLDL, reaching concentrations of 10^1^ ^3^–10^1^ ^5^ particles/mL. Although individual HDL and LDL particles can be as small as ∼10 nm, they readily undergo aggregation in physiological and processing conditions, generating complexes that expand into the 30–100 nm range. This aggregation‐driven size increase results in substantial overlap with the size (30–150 nm) and density (1.02–1.10 g/mL) ranges of extracellular vesicles (EVs), thereby making complete separation by conventional isolation methods exceedingly difficult [5].

Ultracentrifugation (UC) [6] and size‐exclusion chromatography (SEC) [7] remain the most widely used EV isolation methods; however, both exhibit substantial limitations in removing lipoproteins due to these overlapping physicochemical properties [8]. Even combining density‐based (UC) and size‐based (SEC) approaches leaves significant lipoprotein carryover [9, 10]. These impurities pose major challenges: lipoproteins dominate particle counts, confound proteomic/lipidomic profiles, obscure authentic EV signals, and interfere with functional assays by altering uptake kinetics and signaling behavior. Residual lipoproteins further skew biodistribution in therapeutic applications and compromise the sensitivity of diagnostic platforms such as microfluidic and immunoaffinity biosensors [11, 12]. Thus, effective lipoprotein depletion is essential for reliable quantification, molecular profiling, functional studies, and clinical translation of EVs.

Beyond functioning as contaminants, lipoproteins themselves hold biological and clinical importance. ApoA1‐ and ApoB‐positive particles are involved in lipid metabolism, cardiovascular disease, neurodegeneration and serve as potential therapeutic delivery vehicles [13]. Accordingly, technologies enabling selective capture and separation of lipoproteins offer dual benefits: improving EV purity while enabling downstream lipoprotein‐focused investigations.

Aptamers, which are short, chemically stable oligonucleotides with high target specificity, have emerged as powerful tools for selective molecular isolation [14]. Aptamer‐based strategies for capturing ApoA1‐ or ApoB100‐positive lipoproteins have been explored [15, 16, 17], and recent studies have proposed ligand‐assisted depletion approaches to reduce lipoprotein interference in EV workflows. However, existing depletion platforms, including bulk‐phase ligand binding, magnetic bead capture, and membrane‐based adsorption, exhibit inherent limitations. Bulk‐phase approaches often induce nonspecific aggregation, magnetic bead systems rapidly saturate and scale poorly with increasing sample volume, and membrane‐based methods are highly susceptible to pore fouling and clogging when exposed to lipoprotein‐rich samples. These issues collectively lead to reduced EV recovery and restrict the scalability of conventional workflows. Several chromatography‐ and filtration‐based approaches have been reported to reduce lipoprotein interference in EV isolation [18]. ApoFilter differs by functioning as a modular upstream pretreatment for molecularly selective lipoprotein removal that is readily compatible with downstream methods, including TFF‐based workflows [19, 20].

To overcome these limitations, we introduce ApoFilter, a conceptually distinct affinity‐filtration platform engineered to selectively remove (V)LDL and HDL from plasma prior to EV isolation. Rather than relying on bulk‐phase binding, magnetic separation, or membrane adsorption, ApoFilter utilizes a vertically stacked, large‐pore mesh architecture functionalized with ApoB100‐ and ApoA1‐specific aptamers. The ∼20 µm pores prevent clogging and fouling, while gravity‐driven flow across the expanded mesh surface promotes efficient molecular interactions.

The multi‐layered design establishes an extended affinity pathway, ensuring that lipoprotein particles encounter multiple aptamer‐coated interfaces as they pass through the column. This configuration effectively transforms a diffusion‐limited capture process into one driven by repeated convective collisions, resulting in rapid, 1 min depletion of (V)LDL and HDL while preserving EV integrity and supporting large‐volume workflows.

Building on the design principles of the previously validated ExoFilter structure [21], ApoFilter incorporates these aptamer‐functionalized meshes into a modular, high‐throughput unit capable of highly specific lipoprotein removal. The stacked architecture provides a 3D affinity matrix with substantially increased cumulative binding area, enabling efficient, high‐specificity capture without inducing fouling or aggregation.

We demonstrate that ApoFilter removes > 99% of plasma lipoproteins while maintaining—and, when paired with ExoTFF, even enhancing—EV recovery. When integrated upstream of ultracentrifugation, SEC, or ExoTFF, ApoFilter markedly improves sample purity, reproducibility, and downstream analytical performance. This workflow‐compatible and user‐friendly pretreatment module resolves a central bottleneck in EV purification and expands the utility of plasma‐derived EVs for molecular profiling, diagnostics, and therapeutic development.

## 2 Results and Discussion

**FIGURE 1:**
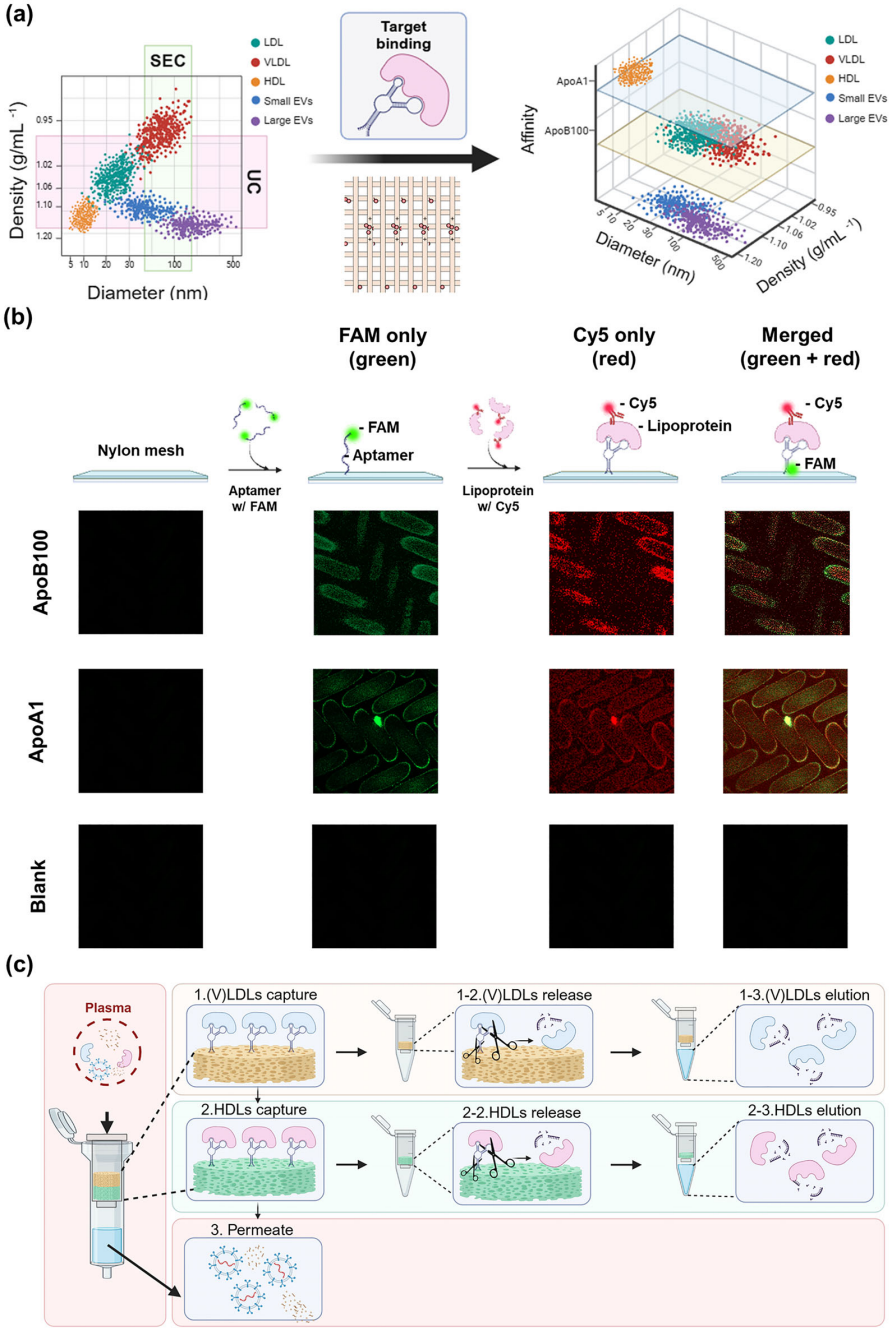
Aptamer‑based affinity filtration enables rapid, selective removal of lipoproteins from plasma EV preparations. (a) Density–size plots of plasma particles illustrate the substantial biophysical overlap between lipoproteins (LDL, VLDL, HDL) and extracellular vesicles (EVs) under conventional ultracentrifugation (UC) and size‐exclusion chromatography (SEC), motivating the need for an affinity‐based separation axis; (b) Fluorescence microscopy shows successful immobilization of FAM‐labeled aptamers (green) onto the nylon mesh and specific binding of Cy5‐labeled (V)LDL and HDL (red), confirming target‐selective molecular recognition. Bare nylon mesh processed without aptamer immobilization (blank control) exhibited negligible fluorescence signals under identical imaging conditions. Green‐only (FAM‐aptamer), red‐only (Cy5‐labeled lipoprotein), and merged images were acquired from the same field of view. (c) Schematic workflow of the sequential capture process: (V)LDL particles are first retained by ApoB100‐specific aptamers in the upper mesh layer, followed by HDL capture via ApoA1 aptamers in the lower layer, yielding a permeate enriched in EVs with lipoproteins efficiently removed.

To address the long‐standing challenge of lipoprotein contamination in extracellular vesicle (EV) isolation from plasma, we developed an aptamer‐based affinity filtration platform, termed ApoFilter, that selectively removes (V)LDL and HDL prior to downstream EV isolation. Lipoprotein contamination remains one of the most persistent obstacles in plasma EV analysis because lipoproteins and small EVs exhibit extensive overlap in both size (30–100 nm) and density (1.02–1.10 g/mL), rendering conventional ultracentrifugation (UC) and size‐exclusion chromatography (SEC) intrinsically insufficient for complete separation (Figure 1a).

To overcome these limitations, several advanced chromatographic strategies incorporating molecular affinity have been developed. In particular, the integrated dual‐mode chromatography platform reported by Van Deun et al. and subsequent refinements represents one of the most sophisticated solutions currently available, combining size‐based and affinity‐based separation to achieve effective lipoprotein depletion [22]. However, such chromatography‐based platforms typically require dedicated instrumentation, complex column preparation, and platform‐specific protocol optimization, which limits their accessibility and routine adoption in many laboratories.

In this context, ApoFilter is not intended to replace these high‐end chromatographic systems. Instead, it is designed as a simple, equipment‐free, and easily adoptable front‐end pretreatment step that lowers the practical barrier to lipoprotein‐depleted EV isolation. By introducing molecular affinity as an additional separation axis through aptamers against ApoB100 and ApoA1—signature markers of (V)LDL and HDL, respectively—ApoFilter enables rapid and selective lipoprotein removal using only a syringe‐driven mesh filtration format. Importantly, this format allows ApoFilter to be readily integrated upstream of diverse downstream EV isolation workflows, including SEC, UC, TFF, and microfluidic platforms.

As detailed in the Methods Section, ApoFilter is constructed from a vertically stacked array of thirty porous nylon meshes separated by spacers that maintain uniform interlayer spacing. Each mesh contains ∼20 µm pores, preventing clogging by nanoscale particles and ensuring consistent gravitational flow. To confer molecular specificity, lipid‐binding aptamers were covalently immobilized onto the mesh surfaces. Fluorescence imaging confirmed successful conjugation: FAM‐labeled aptamers displayed strong co‐localization with Cy5‐labeled (V)LDL and HDL, validating selective recognition and binding fidelity (Figure 1b).

The strategic utility of the ApoFilter architecture lies in its high‐surface‐area vertical stacking, which is critical for maximizing capture efficiency. Unlike conventional batch‐mode systems or column chromatography that rely heavily on the slow, random diffusion of targets, this multi‐layer structure enforces a convective transport mechanism where the entire plasma sample is actively driven through the dense affinity matrix by gravity. The ∼20 µm pores and the short distance between successive aptamer‐functionalized mesh layers are engineered to optimize this forced flow. This design fundamentally transforms the binding kinetics: it maximizes the collision frequency between the abundant lipoprotein targets and the immobilized aptamers, ensuring that virtually every particle encounters a binding site. Consequently, ApoFilter converts the typically diffusion‐limited binding into a rapid, collision‐driven capture process that guarantees a high probability of retention for the target molecules, achieving high removal efficiency even under short residence times (<1 min) and high flow rates.

By integrating ApoB100‐ and ApoA1‐targeting aptamers into the upper and lower layers, respectively, the ApoFilter architecture enables sequential affinity capture of (V)LDL followed by HDL. Operationally, plasma first passes through the ApoB100‐functionalized mesh, where (V)LDL is selectively captured and retained. The permeate then flows into the ApoA1‐functionalized layer for subsequent HDL depletion. The final permeate, now depleted of major lipoproteins while retaining its EV population, is collected for downstream applications requiring high‐purity. To verify the identity of the removed species and confirm binding specificity, the captured lipoproteins can optionally be recovered from the meshes via DNase‐mediated aptamer cleavage (Figure 1c). This modular affinity‐filtration system establishes a 3D separation paradigm—size, density, and molecular recognition—and provides a practical solution for the efficient removal of lipoproteins from complex biofluids such as plasma.

**FIGURE 2:**
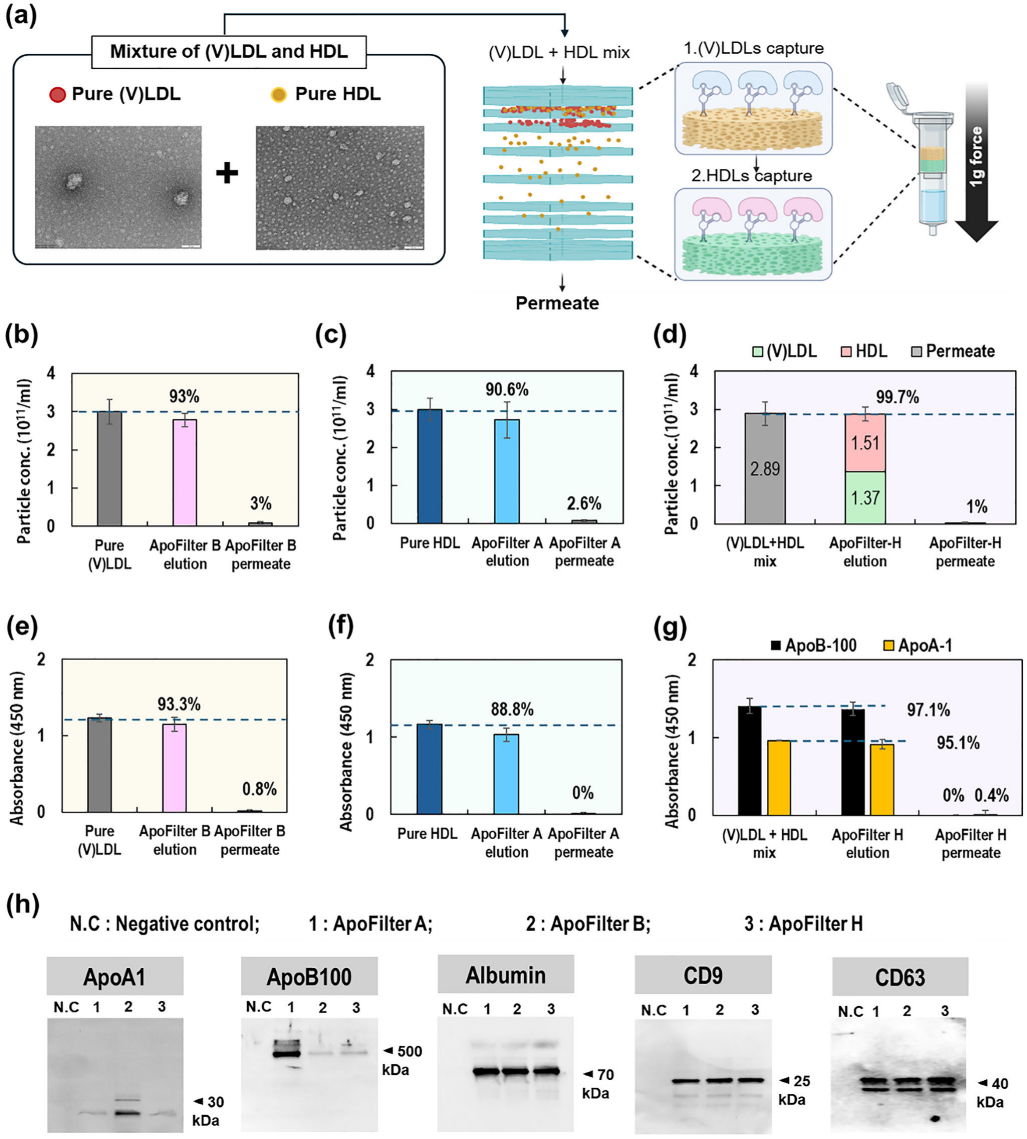
Functional validation of the ApoFilter platform for differential and sequential capture of (V)LDL and HDL (a) Schematic of the hybrid ApoFilter system validated using compositionally pure (V)LDL and HDL preparations; (b–d) Comparative capture efficiency of ApoFilter modules, showing NTA‐quantified retained fractions and residual particles in the permeate for (b) pure (V)LDL, (c) pure HDL, and (d) a mixed (V)LDL+HDL sample; (e–g) ELISA‐based quantification of ApoA1‐ and ApoB100‐positive lipoproteins, illustrating retention efficiency in the capture fractions and residual levels in permeate for (e) pure (V)LDL, (f) pure HDL, and (g) a mixed (V)LDL+HDL sample; (h) Western blot analyses of key protein markers (ApoA1, ApoB100, albumin, CD9, and CD63) in negative control, ApoFilter A, ApoFilter B, and ApoFilter H fractions, demonstrating molecular specificity and integrity of captured and permeate samples.

Figure 2 evaluates the selective lipoprotein‐capture performance of the ApoFilter platform using compositionally pure (V)LDL and HDL suspensions. By employing isolated lipoproteins rather than plasma, the experiment eliminates background nanoparticles and ensures that any particles detected in the permeate directly represent incomplete capture. The purity and identity of each lipoprotein preparation were confirmed by SEM and Western blot prior to filtration. Capture performance was quantified using nanoparticle tracking analysis (NTA) and ELISA under the conditions shown in Figure 2b–e.

NTA measurements demonstrated high removal efficiency across all configurations (Figure 2b–d). ApoFilter‐B retained approximately 93% of (V)LDL, leaving ∼3% in the permeate, while ApoFilter‐A captured 90.6% of HDL with only ∼2.6% remaining. For mixed lipoprotein inputs, the two‐layer system (ApoFilter‐H) achieved > 99% overall depletion, leaving only ∼1% of total particles in the permeate. The enhanced performance of ApoFilter‐H likely arises from the reduced flow velocity across the stacked meshes in this gravity‐driven system, which increases residence time and promotes more efficient target–aptamer interactions. These results confirm that ApoFilter supports robust, selective removal under both single‐target and competitive mixed‐lipoprotein conditions.

As shown in Figure 2e–g, ELISA analysis robustly validated the molecular specificity and efficiency of aptamer‐mediated capture across all conditions. For pure (V)LDL samples (Figure 2e), ApoFilter‐B retained 93.3% of ApoB100‐positive (V)LDL, with only 0.8% remaining in the permeate. For pure HDL samples (Figure 2f), ApoFilter‐A retained 88.8% of ApoA1‐positive HDL, with no detectable signal in the permeate. In the mixed (V)LDL+HDL condition (Figure 2g), ApoFilter‐H exhibited highly efficient and parallel depletion, retaining 97.1% of ApoB100‐positive (V)LDL and 95.1% of ApoA1‐positive HDL. The corresponding permeate fractions contained only 0% (V)LDL and 0.4% HDL, confirming nearly complete and highly selective removal of both lipoprotein species in the presence of competitors.

To further validate the molecular specificity and performance of the ApoFilter platform, we performed Western blot analysis of key protein markers using permeate fractions collected after passage through ApoFilter A, ApoFilter B, or the hybrid ApoFilter H (Figure 2h). ApoA1 and ApoB100 were markedly depleted or undetectable in all permeate samples following their respective filtration steps, confirming highly efficient and selective removal of HDL and (V)LDL from the processed plasma. Albumin, a major plasma protein, was consistently detected across all permeate fractions, demonstrating that ApoFilter preserves non‐target proteins and does not induce nonspecific loss. Critically, canonical EV markers (CD9, CD63) remained abundant in the permeate after ApoFilter treatment, indicating that EV integrity and recovery are maintained throughout the filtration process. These results—obtained exclusively from ApoFilter‐permeate samples—provide robust protein‐level evidence for the platform's selectivity, supporting its utility for high‐purity EV isolation from lipoprotein‐rich plasma.

**FIGURE 3:**
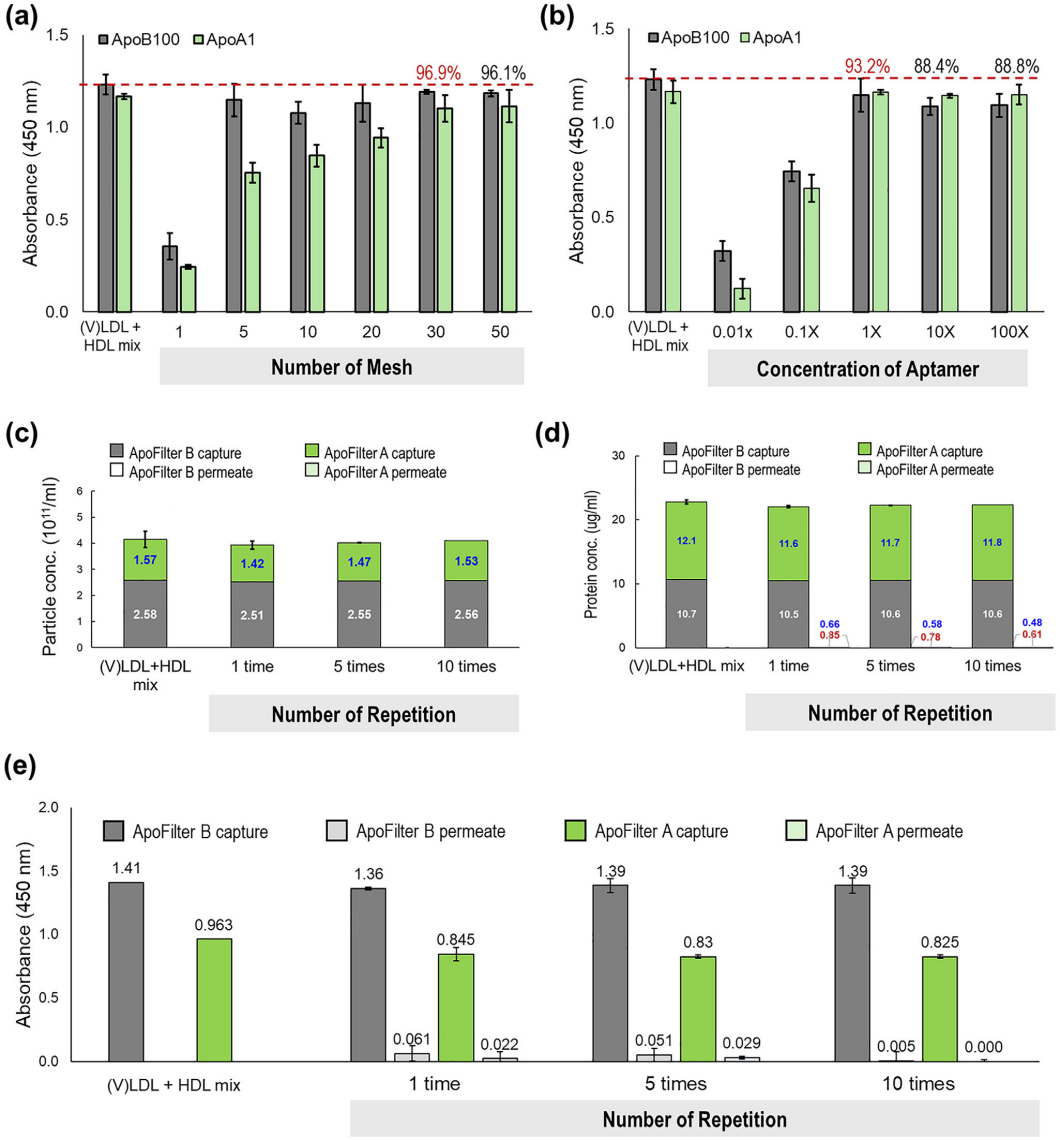
Optimization of ApoFilter operational parameters to enhance lipoprotein capture performance. (a) Effect of mesh layer number on the recovery of (V)LDL and HDL from mixed samples; (b) Influence of aptamer loading concentration on capture efficiency under fixed mesh area; (c–e) Evaluation of repeated gravity‐driven filtration cycles, showing that while multiple recirculation further decrease residual lipoproteins, a single filtration pass already achieves near‐maximal depletion across NTA, BCA, and ELISA measurements.

These results demonstrate that aptamer‐mesh consistently maintains high affinity for its target, enabling selective capture and removal even under competitive conditions. This establishes ApoFilter as a highly selective affinity‐filtration platform capable of efficient (V)LDL and HDL depletion in various lipoprotein systems. Subsequent optimization studies (Figure 3) further refine the platform's structural and operational parameters—including mesh layer number, aptamer loading concentration, and filtration cycling—to achieve consistently near‐complete lipoprotein removal under practical operating conditions.

Sequential optimization began by evaluating the number of mesh layers required for aptamer immobilization under a mixed input of (V)LDL (1 mL) and HDL (1 mL), each at a concentration of 20.7 mm (HDL) and 12.90 mm ((V)LDL) (Figure 3a). Given that each mesh disk has a diameter of 5 mm and a thickness of 0.1 mm, the available surface area per layer can be estimated accordingly. Increasing the number of mesh layers progressively improved lipoprotein removal. For (V)LDL, ApoFilter‐B achieved near‐maximal capture with only five layers (93.2%), whereas HDL depletion continued to improve with additional layers, reaching optimal performance at 30 layers. These results indicate that expanding the affinity surface area enhances capture kinetics by increasing the probability of contact between lipoproteins and immobilized aptamers. Although this mesh configuration was optimal for the tested volume, larger input volumes would likely require proportionally greater filtration surface area, achievable by increasing the housing diameter or adding more mesh layers.

While total binding area is a critical parameter, we reasoned that the effective aptamer density on the mesh would also influence capture performance. Because direct quantification of immobilized aptamers was not performed, we instead varied the concentration of aptamer supplied during the conjugation reaction while keeping the total surface area constant at 30 mesh layers (Figure 3b). Higher aptamer concentrations yielded progressively improved depletion, with > 93% removal at the highest concentration tested. Notably, even the baseline concentration (1X = 1.16 um (HDL) and 0.7 mm ((V)LDL)) produced substantial capture, and further increases resulted in only incremental gains. These findings indicate that using the optimal amount of aptamer during the conjugation process enhances the ligand (aptamer) density per unit surface area, thereby enabling more effective capture of lipoprotein targets.

To further investigate factors affecting capture efficiency, we examined the impact of repeated filtration cycles (Figure 3c–e). Because flow through the stacked mesh occurs solely by gravity, lipoproteins experience limited residence time on the aptamer‐functionalized surfaces. Under these conditions, approximately 1 mL of pure (V)LDL or pure HDL passed through 30 layers in < 5 min (∼0.2 mL min^−^^1^). We hypothesized that this short contact time might prevent binding equilibrium from being reached during a single pass; therefore, filtration was repeated five and ten times to increase cumulative interaction.

As shown in Figure 3c–e, even with repeated processing, only a slight reduction in permeate particle counts was observed, and any increase in captured particles was minimal. Notably, a single filtration cycle was sufficient to achieve nearly maximal removal efficiency—over 97% for isolated lipoprotein samples and over 98% for mixed samples. These findings indicate that aptamer–target interactions occur rapidly and efficiently within the brief contact time of a single pass, and that extended recirculation provides only limited additional benefit.

Until now, we have evaluated the standalone performance of ApoFilter in removing lipoproteins from pure lipoprotein solutions. In the following section, we assess its performance in human plasma, which represents the most complex biological matrix, comprising diverse proteins, ions, extracellular vesicles (EVs), and lipoproteins.

**FIGURE 4:**
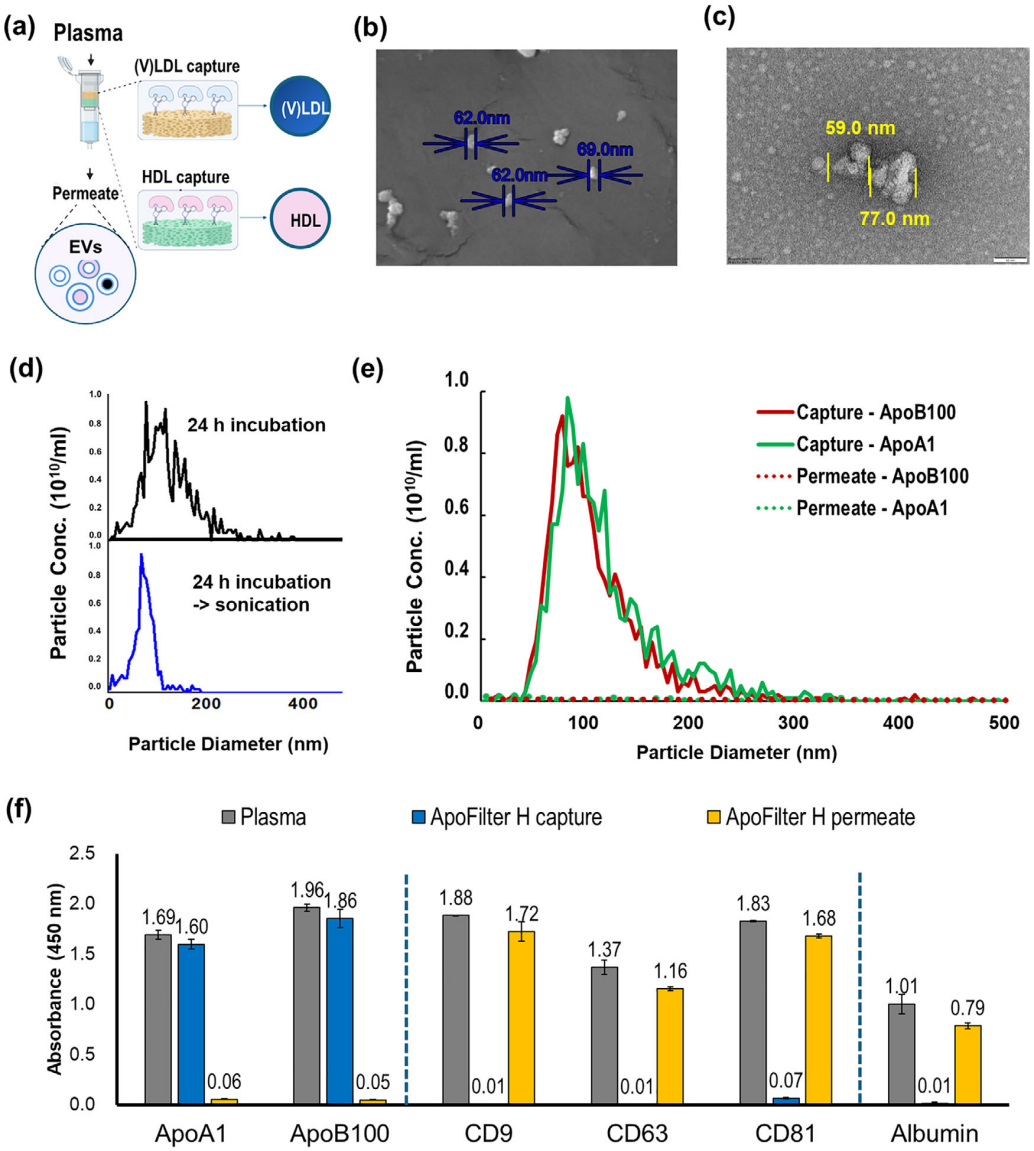
Specificity and performance validation of the ApoFilter system for extracellular vesicle isolation from complex human plasma. (a) Schematic illustration of the ApoFilter workflow, depicting sequential gravity‐driven capture of (V)LDL and HDL and collection of EV‐enriched permeate; (b,c) SEM and TEM imaging analyses, revealing nanoscale aggregation states and morphological heterogeneity of plasma‐derived lipoproteins, highlighting their relevance for downstream fluorescence‐based assays; (d) Optimization of dispersion conditions, showing that extended incubation induces lipoprotein aggregation, while brief sonication restores expected nanoparticle size distributions; (e) Fluorescence‐NTA analysis of plasma spiked with Alexa Fluor–labeled anti‐ApoB100 and anti‐ApoA1 antibodies confirms near‐complete removal of fluorescently labeled lipoproteins following ApoFilter processing, with dotted traces indicating the permeate fractions; (f) ELISA quantification of ApoA1 and ApoB100, demonstrating efficient depletion of HDL and (V)LDL, with preservation of EV markers (CD9, CD63, CD81) and albumin recovery.

Plasma samples, serving as the target biological matrix, were directly processed using ApoFilter‐H, designed for sequential capture of (V)LDL and HDL (Figure 4a). The overall workflow and filtration sequence are shown in Figure 4a, depicting gravity‐driven passage of plasma through stacked, aptamer‐functionalized mesh layers, which enables selective removal of lipoproteins while collecting EV‐enriched permeate.

To clarify the morphological states of lipoproteins in plasma and exclude purification‐induced artifacts, we performed electron microscopy. In native plasma, lipoproteins rapidly assembled into nanoscale aggregates, as visualized by SEM and TEM (Figure 4b,c), consistent with their known colloidal instability in protein‐rich environments. This intrinsic propensity for aggregation can significantly distort particle size distributions obtained by conventional NTA analysis.

As illustrated in Figure 4d, pure lipoprotein samples are equally prone to aggregation during standard handling and labeling procedures, resulting in NTA profiles mimicking those of EVs. When fluorescently labeled lipoproteins were incubated for 24 hours, the measured size profile shifted markedly toward larger hydrodynamic diameters—a hallmark of aggregation. Gentle sonication dispersed these aggregates, restoring the expected sub‐100 nm population and confirming that aggregation is reversible and highly sensitive to sample‐handling parameters, such as incubation duration and dispersion energy. Notably, this technical challenge is particularly relevant for fluorescence‐based NTA analysis, which typically requires extended incubation for dye‐based labeling and may therefore amplify aggregation artifacts, even in pure lipoprotein suspensions.

Fluorescence‐NTA analysis further confirmed that fluorescently labeled lipoproteins were efficiently removed by ApoFilter, while the EV population profile was preserved (Figure 4e). In the captured fractions recovered from ApoFilter, particle size and concentration distributions closely resembled those of the input, with strong fluorescent signals corresponding to ApoA1‐ and ApoB100‐positive particles. In sharp contrast, the permeate exhibited fluorescence levels approaching the instrument's detection limit across the size range, demonstrating that virtually no labeled lipoproteins passed through the filter. This clear separation between capture and permeate fractions confirms that ApoFilter enables highly efficient and selective depletion of lipoproteins from plasma without perturbing the EV size distribution.

Finally, ELISA analysis demonstrated efficient depletion of ApoA1‐ and ApoB100‐positive lipoproteins (0.06/1.69 and 0.05/1.96 for capture/permeate, respectively; Figure 4f). Meanwhile, canonical EV markers (CD9, CD63, CD81) and albumin levels remained largely unchanged. This indicates that the affinity filtration process selectively removes lipoproteins without compromising EV integrity or depleting major plasma proteins, preserving physiologically relevant sample composition for downstream assays.

Collectively, these findings validate that ApoFilter functions through specific affinity‐based retention rather than nonspecific adsorption or size‐based trapping and underscore its suitability for processing complex plasma samples, as well as seamless integration into downstream EV isolation and analysis workflows.

**FIGURE 5:**
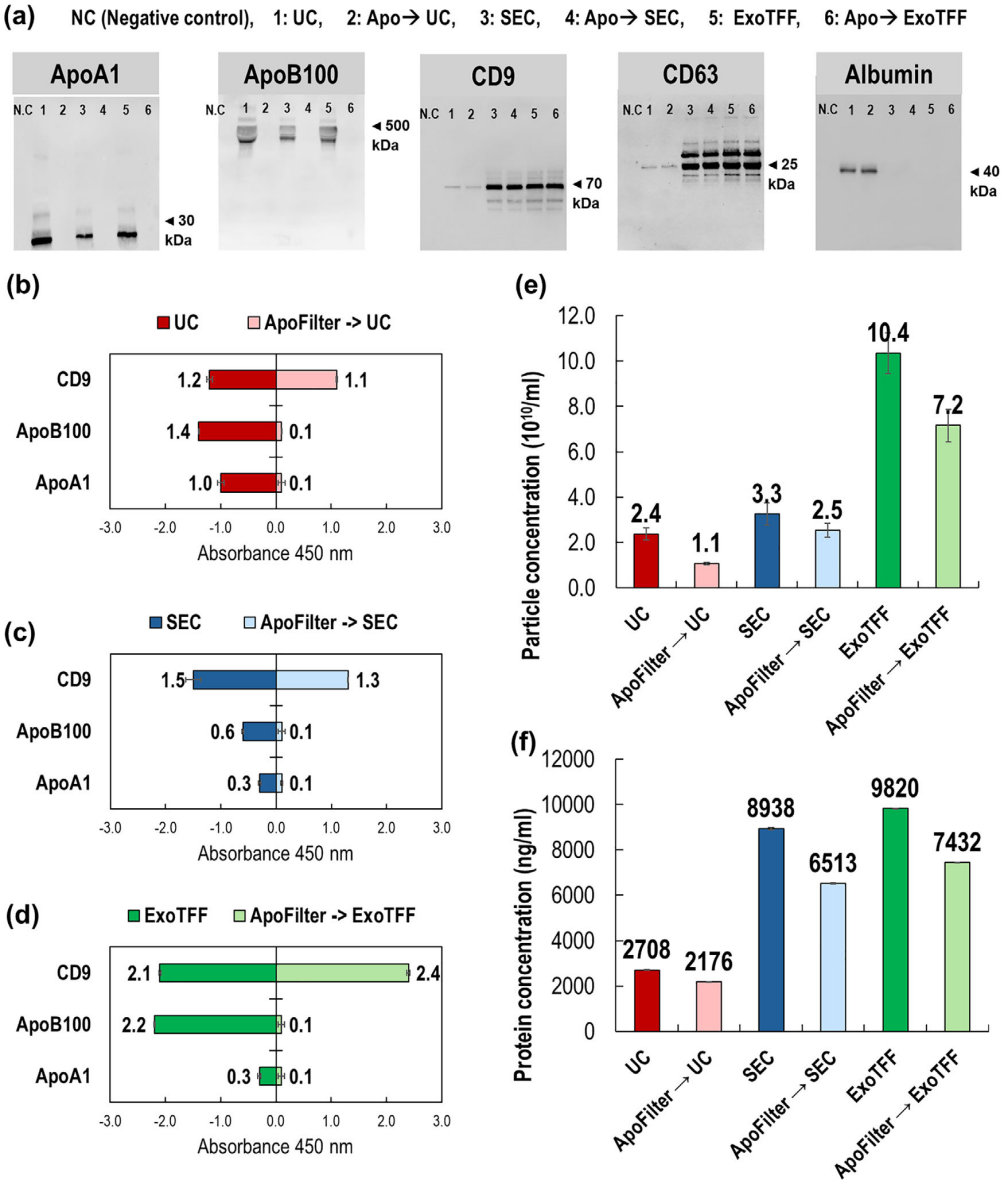
Synergistic removal of lipoproteins and preservation of EV yield by integrating ApoFilter with standard EV isolation workflows. (a) Western blot analysis of ApoA1 and ApoB100 (lipoprotein markers), CD9 and CD63 (EV markers), and albumin across samples isolated by ultracentrifugation (UC), size‐exclusion chromatography (SEC), or ExoTFF, with (+) or without (–) ApoFilter pretreatment; (b–d) ELISA‐based quantification of ApoA1, ApoB100, and CD9 demonstrating enhanced lipoprotein depletion and preserved EV markers across each workflow following ApoFilter pretreatment; (e) Nanoparticle tracking analysis (NTA) showing particle concentrations for each isolation method, highlighting the reduction of non‐EV particles upon ApoFilter integration; (f) Total protein concentration measured by BCA assay, showing reduced protein loads across workflows incorporating ApoFilter, consistent with efficient lipoprotein removal rather than albumin depletion.

To further assess the utility of ApoFilter in practical EV workflows, we next evaluated its performance when integrated with widely used EV isolation methods, examining both its lipoprotein‐depletion capability and its impact on EV recovery. Three established techniques were selected for comparison: UC, SEC, and ExoTFF. ExoTFF, recently developed by our group, combines electrokinetic principles derived from the ExoFilter platform with TFF, allowing particles below a defined cutoff to permeate while retaining EVs. Plasma samples were processed under six conditions to compare the performance of the combined workflows (Figure 5).

Western blot analysis of permeate fractions (Figure 5a) revealed clear differences in lipoprotein content depending on whether ApoFilter pretreatment was used. Strong ApoA1 and ApoB100 bands persisted in UC, SEC, and ExoTFF when performed alone, whereas both markers were sharply reduced or eliminated following ApoFilter application. By contrast, EV markers (CD9, CD63) and albumin displayed comparable band intensities with or without ApoFilter, indicating that the filtration step selectively removes lipoproteins without depleting EVs or abundant plasma proteins.

Quantitative ELISA analysis further resolved the performance differences across workflows (Figure 5b–d). For UC, substantial amounts of ApoB100 (1.4) and ApoA1 (1.0) remained after isolation, demonstrating incomplete lipoprotein removal. With ApoFilter pretreatment, both markers dropped to 0.1, indicating near‐complete depletion, while CD9 retention remained high (1.2 → 1.1; 91.7%).

For SEC, ApoB100 and ApoA1 were partially reduced (0.6 and 0.3, respectively), consistent with SEC's known capability for HDL removal but limited LDL clearance. ApoFilter pretreatment again reduced both markers to 0.1, with a modest decrease in CD9 (1.5 → 1.3; 86.7% retention). ExoTFF alone achieved strong EV recovery (CD9 = 2.1) but poor lipoprotein removal (ApoB100 = 2.2). When ApoFilter was applied upstream, ApoB100 and ApoA1 were reduced to 0.1, and notably, CD9 increased from 2.1 to 2.4. We attribute this enhancement to the selective removal of negatively charged lipoproteins, which reduces electrostatic competition within the ExoTFF module and increases the effective interaction surface available for EV retention. While these results demonstrate the compatibility of ApoFilter with multiple EV isolation workflows under our experimental conditions, future multi‐center or cross‐laboratory studies will be valuable to further evaluate the generalizability of ApoFilter‐integrated EV isolation workflows.

These results highlight intrinsic differences among the isolation strategies: SEC provides the highest HDL‐depletion efficiency, ExoTFF achieves the strongest EV recovery albeit with limited LDL clearance, and UC performance is the most variable owing to operator dependence. Importantly, across all three methods, ApoFilter pretreatment enabled near‐complete removal of both HDL and LDL while preserving—or in the case of ExoTFF, enhancing—EV yield. Thus, ApoFilter serves as a broadly compatible upstream module that strengthens existing EV workflows by minimizing lipoprotein interference without compromising EV integrity.

Nanoparticle tracking analysis (Figure 5e) further supported this conclusion. ApoFilter pretreatment consistently reduced particle counts across all workflows: by 54% for UC (2.4 × 10^10^ → 1.1 × 10^10^ particles/mL), 24% for SEC (3.3 × 10^10^ → 2.5 × 10^10^ particles/mL), and 31% for ExoTFF (10.4 × 10^10^ → 7.2 × 10^10^ particles/mL). Since lipoproteins are abundant nanoparticles within plasma, these reductions primarily reflect the removal of non‐EV contaminants that would otherwise be miscounted as EVs.

Finally, total protein assessment by BCA assay (Figure 5f) revealed a parallel trend: ApoFilter pretreatment uniformly lowered protein concentrations relative to non‐filtered controls. Given the protein‐rich nature of lipoproteins, this pattern aligns with their depletion and confirms that the majority of removed mass originates from lipoproteins rather than EVs. Together, these results demonstrate that ApoFilter enhances sample purity, improves the interpretability of EV quantification, and maintains EV recovery across diverse isolation platforms.

**FIGURE 6:**
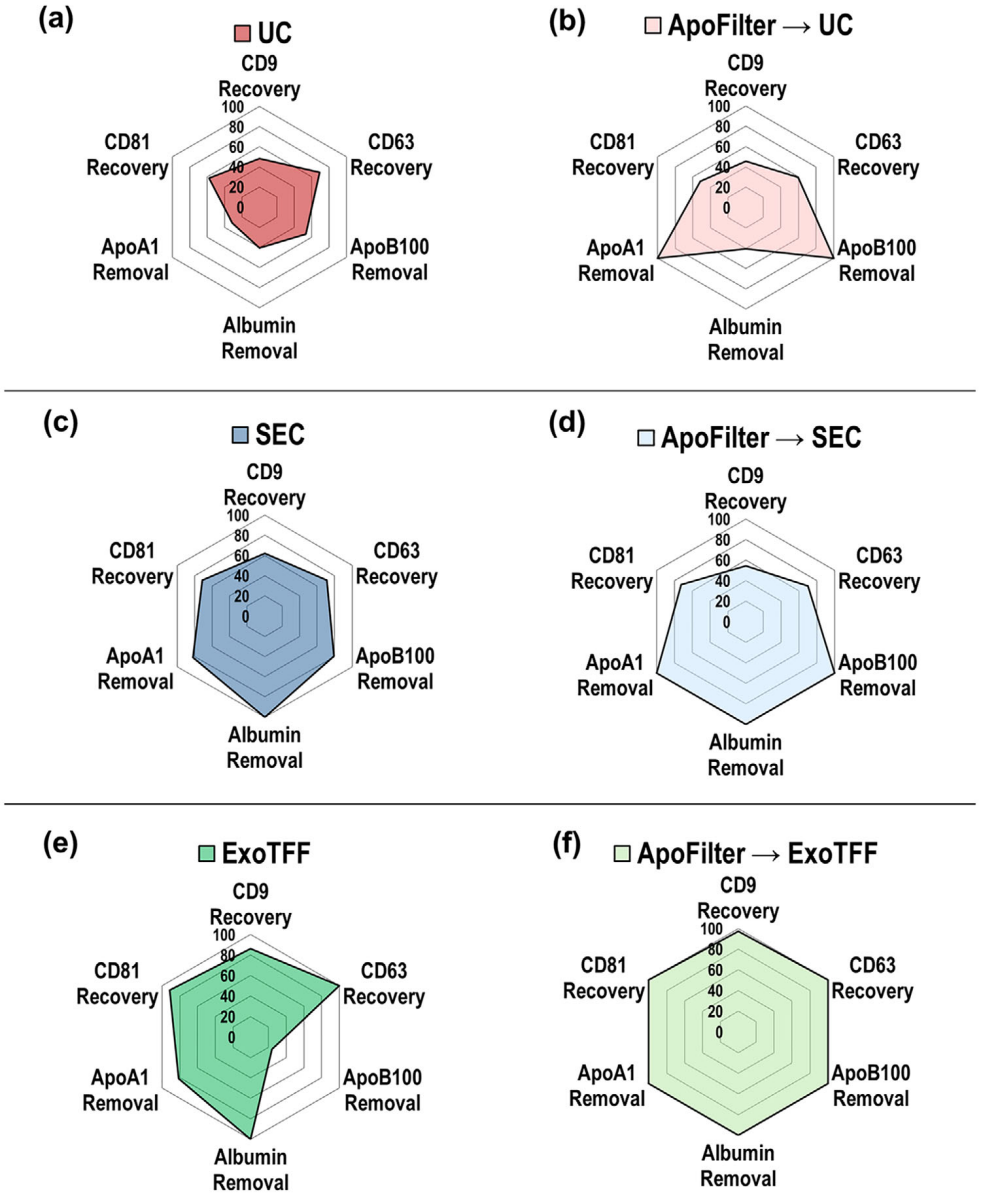
Performance comparison of EV isolation workflows with and without ApoFilter pretreatment. Radar plots depict percentage recovery of EV markers (CD9, CD63, CD81) and removal of contaminant proteins (ApoA1, ApoB100, albumin) for each method: (a) UC, (b) ApoFilter→UC, (c) SEC, (d) ApoFilter→SEC, (e) ExoTFF, and (f) ApoFilter→ExoTFF. Integration of ApoFilter markedly increases lipoprotein (ApoA1/ApoB100) depletion while maintaining or enhancing EV marker recovery across all workflows.

Figure 6 provides a comparative overview of three commonly used EV isolation methods—ultracentrifugation (UC), size‐exclusion chromatography (SEC), and ExoTFF—applied alone or in combination with ApoFilter, using plasma samples. Although plasma values are not visually presented in Figure 4b–d, the original absorbance values of plasma samples were measured in parallel and served as reference points for calculating the removal efficiency of lipoproteins (ApoB100, ApoA1) and the recovery efficiency of EV markers (CD9). These calculated values allow a more accurate comparison of each isolation strategy's performance and are visualized in the radar plots of Figure 5. EV recovery was assessed using three canonical EV markers (CD9, CD63, and CD81), while purification performance was evaluated based on the depletion of major plasma contaminants, including lipoproteins (ApoA1, ApoB100) and albumin. The radar plots clearly illustrate the trade‐offs and synergistic effects across the evaluated workflows.

UC alone demonstrated modest EV recovery but poor impurity removal, with particularly low depletion rates for lipoproteins and albumin. Upon integration with ApoFilter, however, lipoprotein removal markedly improved, while EV recovery remained largely unaffected. SEC, in contrast, achieved relatively better performance in albumin removal and moderate lipoprotein depletion, albeit with slightly lower EV recovery. When combined with ApoFilter, SEC exhibited near‐complete removal of both lipoproteins and albumin, with only a marginal impact on EV yield.

ExoTFF showed limited capacity to remove larger lipoproteins such as (V)LDL. Remarkably, the combination of ApoFilter with ExoTFF not only enabled efficient removal of both (V)LDL as well as HDL, but also enhanced EV recovery, highlighting a synergistic benefit. Collectively, these findings underscore the value of integrating ApoFilter into EV isolation workflows to simultaneously enhance purity and yield, especially in plasma samples where lipoprotein contamination poses a significant analytical challenge.

In this study, we developed and validated an aptamer‐based affinity filtration technology (ApoFilter) designed to resolve a long‐standing bottleneck in extracellular vesicle (EV) isolation: the pervasive co‐isolation of lipoproteins from blood. Using both compositionally pure lipoprotein preparations and human plasma, we demonstrated that ApoFilter achieves highly selective and efficient removal of (V)LDL and HDL, routinely exceeding 99% depletion. This led to markedly improved EV purity across all tested workflows, with the most pronounced enhancement observed when ApoFilter was integrated with ExoTFF (Figure 5).

A central advantage of ApoFilter lies in its operational simplicity and ultrafast kinetics. Whereas conventional immunoaffinity systems require incubation periods exceeding one hour, ApoFilter achieves comparable or superior target capture during brief, gravity‐driven filtration lasting <1 min. This rapid binding can be attributed to several synergistic design elements. First, the ApoB100‐ and ApoA1‐targeting aptamers used in this study possess sub‐nanomolar affinity, enabling rapid, near‐diffusion‐limited complex formation upon molecular contact [23]. Second, the mesh‐based format [21, 24], forces convective flow through a densely functionalized surface, transforming a diffusion‐limited binding process into a collision‐driven capture mechanism that greatly increases the frequency of aptamer–lipoprotein interactions. Finally, the multi‐layer mesh architecture expands the effective binding surface area by several orders of magnitude, ensuring that transient passage through the filter is sufficient to achieve near‐complete depletion. Collectively, these features convert traditional immunoaffinity capture—typically slow and batch‐limited—into a rapid, continuous‐flow process with high throughput and excellent reproducibility.

ApoFilter also compares favorably to recently reported lipoprotein‐depletion strategies. For example, A recent study [19] described a ligand‐assisted negative‐selection method, but that approach relies on solution‐phase bead capture followed by centrifugation‐based separation—steps that can induce aggregation, increase nonspecific loss, and reduce EV recovery. In contrast, ApoFilter operates through surface‐anchored affinity capture on a porous mesh, eliminating pellet‐recovery steps and minimizing sample loss. Moreover, our dual‐layer configuration enables sequential depletion of (V)LDL and HDL, while preserving EV integrity and yield, distinguishing ApoFilter conceptually and functionally from previously reported systems.

When integrated with existing EV isolation workflows—including UC, SEC, and ExoTFF—ApoFilter served as an effective upstream purification module. Although UC and SEC displayed only modest improvements in EV recovery following lipoprotein removal, both benefited substantially in terms of purity. By contrast, ExoTFF showed synergistic enhancement: ApoFilter pretreatment not only reduced lipoprotein contamination to near‐background levels but also increased EV recovery from 85.4% to 98.2%. We attribute this improved yield to the removal of similarly sized, negatively charged lipoproteins, which otherwise compete with EVs during the electrokinetic capture step of ExoTFF [24]. This observation highlights the unique complementarity between affinity‐based pre‐depletion and electrokinetic isolation, a combination that has not been demonstrated previously.

From a practical perspective, cost is an important consideration for the adoption of any EV isolation workflow. Although a detailed cost analysis is beyond the scope of this study, ApoFilter is conceptually designed to rely on synthetic DNA aptamers and simple polymeric components, which are, in principle, amenable to scalable and cost‐efficient manufacturing. Only a small amount of aptamer is required per mesh due to surface immobilization, and the remaining components consist of inexpensive, non‐specialized materials. We also note that combining ApoFilter with downstream methods such as size‐exclusion chromatography (SEC) will inevitably increase the overall workflow cost. In this regard, ApoFilter is intended as a modular pretreatment step that can be used either as a stand‐alone lipoprotein depletion module or integrated into more stringent purification workflows, allowing users to balance cost, workflow complexity, and EV purity requirements depending on the application.

Despite its strong performance, this study has several limitations. The present work focuses primarily on plasma, where lipoproteins are uniquely abundant and represent a major obstacle to EV isolation. The current ApoFilter design already addresses the major plasma lipoprotein classes such as HDL, LDL, and VLDL through combined ApoA1 and ApoB100 targeting. In contrast, other lipoprotein subclasses, such as ApoE‐enriched remnant particles, constitute a relatively minor subpopulation in plasma and were not included in the present study in order to maintain a focused and practical system design. Future work may extend the platform to incorporate additional affinity reagents targeting such specialized lipoprotein subclasses or other plasma contaminants, depending on specific clinical or analytical needs.

While we confirmed preservation of key EV markers and recovery, future work should rigorously evaluate whether ApoFilter‐processed EVs retain their native biological functions, including cellular uptake, molecular signaling, and immunomodulatory activity, in contexts directly relevant to clinical diagnostics and therapeutics. Robust validation in EV‐based biomarker detection, patient stratification, and longitudinal clinical monitoring will be essential for full clinical translation.

To further address the current limitations of negative‐selection affinity platforms, our ongoing research is focused on the development of TetraFilter, an advanced system leveraging positive affinity capture. Unlike ApoFilter, which selectively removes lipoproteins, TetraFilter directly targets EV‐specific markers such as CD9 and CD63, enabling selective capture and isolation of functionally intact EV subpopulations from complex plasma samples. We anticipate that this approach will offer even greater purity and specificity, facilitating downstream molecular analyses and clinical applications.

Expansion of the aptamer library to target additional plasma contaminants, as well as translational deployment using standardized protocols and automated processing units for high‐throughput workflows, will further pave the way toward next‐generation EV‐based diagnostics and therapeutics. We are confident that continued interdisciplinary research and technological innovation will fully realize the translational potential of these platforms.

In conclusion, we present ApoFilter as a rapid, selective, and broadly compatible affinity filtration technology that addresses one of the most persistent challenges in EV isolation. By enabling near‐complete lipoprotein removal without compromising EV integrity, ApoFilter substantially improves the purity, reproducibility, and interpretability of EV analyses. This advancement is poised to enhance EV‐based liquid biopsy applications, supporting earlier disease detection, precision diagnostics, and therapeutic monitoring. Continued refinement and clinical translation of ApoFilter may further position this platform as a robust, scalable solution for next‐generation EV research and diagnostics.

## 3 Materials and Method

### 3.1 Design of the Aptamer

Aptamers targeting ApoB‐100 and ApoA‐1 were synthesized with a 3′‐terminal C6‐NH_2_ modification (Bioneer, Daejeon, Korea).

The sequences used in this study were as follows:

**Table:** 

Target	Sequence (5’ → 3’)	Ref.
ApoB‐100	5'—ACCT CGAT TTTA TATT ATTT CGCT TACC AACA ACTG CAGA ‐C6‐NH _2_ 3'	[16]
ApoA‐1	5'‐CCTC GGCA CGTT CTCA GTAG CGCT CGCT GGTC ATCC CACA‐C6‐NH _2_ 3'	[17]

### 3.2 Aptamer Functionalization of Nylon Mesh

Nylon meshes (11 mm diameter; Lixin Huarun Mesh Co.) were activated in 0.1 N HCl for 30 min, thoroughly rinsed with deionized water, and subsequently treated with 2.5% glutaraldehyde. For aptamer conjugation, 1.4 µg of aptamer was incubated with the activated meshes in an EDC/NHS coupling solution prepared in 0.9% NaCl for 6 h at room‐temperature. After conjugation, the functionalized meshes were extensively washed and stored in PBS at 4°C until use. Detailed step‐by‐step fabrication procedures, including reagent concentrations, incubation conditions, washing steps, and assembly parameters, are provided in (Section S2).

### 3.3 Sample Processing

Human plasma (Zen‐Bio) was centrifuged (3000 × g, 15 min) and filtered (800 nm). Pure (V)LDL and HDL (Merck KGaA) were handled identically. All samples were aliquoted and stored at −80°C. Human samples complied with the Declaration of Helsinki.

### 3.4 Microscopy (Confocal / SEM / TEM)

#### 3.4.1 Confocal Microscopy

Aptamer‐FAM and Cy5‐lipoprotein colocalization was imaged using a Zeiss LSM800. Images were processed using ZEN Black and FIJI (JACoP plugin).

#### 3.4.2 Scanning Electron Microscopy (SEM)

EVs captured on AAO membranes were fixed, ethanol‐dehydrated, Pt‐coated, and imaged using Quanta 250 FEG (FEI).

#### 3.4.3 Transmission Electron Microscopy (TEM)

Lipoproteins were adsorbed on Formvar‐coated grids, stained with 1% uranyl acetate, and imaged using JEM‐1400 Flash (JEOL).

### 3.5 Nanoparticle Tracking Analysis (NTA)

EVs and lipoproteins were analyzed using ZetaView (Particle Metrix). Dilution ensured 140–200 particles/frame. Data were collected at 11 positions × 60 frames with standard EV settings. Analysis used ZetaView software v8.02.31.

### 3.6 Fluorescent Labeling of Captured Lipoproteins

To visualize and verify the selective capture of lipoproteins during plasma processing, fluorescent labeling was performed using exogenously added antibodies. Alexa Fluor 647–conjugated anti‐ApoB100 and Alexa Fluor 488–conjugated anti‐ApoA1 antibodies (R&D Systems) were added to the elution samples at a 1:10 dilution and incubated for 24 h at 37°C under gentle agitation.

This spike‐in labeling strategy was used solely for fluorescence‐based visualization and quantification of the removed lipoproteins and was not required for routine EV purification. Corresponding permeate fractions were processed in parallel as negative controls.

### 3.7 Protein Quantification (BCA)

Protein levels were measured using the Pierce BCA kit. Absorbance at 562 nm was measured using a DS‐11 spectrophotometer.

### 3.8 Western Blot

Samples were prepared in Laemmli buffer, separated via SDS‐PAGE, and transferred to PVDF membranes. Primary antibodies: CD9, CD63, CD81, ApoA1, ApoB100, albumin. Signals were detected using HRP‐conjugated secondary antibodies and ECL (ChemiDoc XRS+).

### 3.9 Immunocapture ELISA

Anti‐CD9, CD63, CD81, ApoB100, or ApoA1 capture antibodies (5 µg/mL) were immobilized in 96‐well plates. Samples and detection antibodies were incubated sequentially, followed by streptavidin‐HRP and TMB substrate. Absorbance was measured at 450 nm (SPECTROstar Nano).

### 3.10 EV Isolation Methods

#### 3.10.1 Ultracentrifugation (UC)

Human plasma was centrifuged (3000 × g; 12 000 × g), then ultracentrifuged at 120 000 × g for 2 h (Hitachi CP100WX). Pellets were washed and resuspended in PBS.

#### 3.10.2 Size‐Exclusion Chromatography (SEC)

Human plasma (900 µL) was loaded onto qEVoriginal 70 nm columns (Izon). EV‐enriched fractions (F8–F10) were collected and pooled.

### 3.11 ExoTFF

A syringe‐type ExoFilter captured EVs on positively charged meshes while TFF removed sub‐30 nm proteins and lipoproteins. EVs were eluted using 1 m NaCl and recovered into 2 mL PBS.

## Author Contributions

S.Y.J. contributed formal analysis, investigation, methodology, writing – original draft; Y.W.K contributed investigation, methodology; S. S. contributed conceptualization; formal analysis; methodology; supervision; visualization; writing – review & editing.

## Conflicts of Interest

The authors declare no conflict of interest.

## Supporting information




**Supporting Information**: smll73023‐sup‐0001‐SuppMat.docx.

## Data Availability

The main data supporting the results of this study are available within the manuscript and supplementary information files. The raw data files are available for research purposes from the corresponding author upon reasonable request. Source data are provided with this paper.
