# Supplementary material for: Selective Lipoprotein Removal Enables High‐Purity EV Isolation from Plasma via Aptamer‐Based Mesh Filtration
Source: Small. 2026 Mar 3;22(24):e14724. doi: 10.1002/smll.202514724 (PMC13114492; doi:10.1002/smll.202514724)
Supplement: Supplementary file 1 — Supporting Information: smll73023‐sup‐0001‐SuppMat.docx. [file SMLL-22-e14724-s001.docx]

**Supplementary Information for**

**Selective Lipoprotein Removal Enables High-Purity EV Isolation from Plasma via Aptamer-Based Mesh Filtration**

Soyoung Jeon^1^, YongWoo Kim^2^, Sehyun Shin ^1,2,3*^

^1^ Department of Micro-Nanosystem Technology, Korea University, Seoul, Korea

^2^ School of Mechanical Engineering, Korea University, Seoul, Republic of Korea

^3^ Engineering Research Center for Biofluid Biopsy, Seoul, Korea

* To whom correspondence should be addressed:

Sehyun Shin, Ph.D.

Professor,

School of Mechanical Engineering, Korea University, Seoul 02841, Republic of Korea;

Tel.: +82 2 3290 3377; Fax: +82 2 928 5825; E-mail: lexerdshin@korea.ac.kr

ORCID ID: 0000-0002-2611-5610

## S1. Aptamer Sequences and Design

All reagents were analytical grade and used as received. Aptamers (ApoB100, ApoA1) were synthesized by Bioneer (Daejeon, Korea). Nylon mesh was sourced from Lixin Huarun MESH Co. Pure (V)LDL and HDL were obtained from Merck KGaA. Human plasma was purchased from Zen-Bio Inc.

Two distinct DNA aptamers targeting apolipoprotein markers were used in this study. All aptamers were synthesized with a 3′ C6-NH₂ modification (Bioneer, Daejeon, Korea) to facilitate covalent attachment to nylon mesh surfaces.

*S1.1 Aptamer Sequences*

| Target | Sequence (5’ → 3’) | Ref. |
| --- | --- | --- |
| ApoB-100 | 5' - ACCT CGAT TTTA TATT ATTT CGCT TACC AACA ACTG CAGA -C6-NH _2_ 3' | ^[16]^ |
| ApoA-1 | 5'-CCTC GGCA CGTT CTCA GTAG CGCT CGCT GGTC ATCC CACA-C6-NH _2_ 3' | ^[17]^ |

Aptamers were resuspended in nuclease-free water (Thermo Fisher) at 100 μM, aliquoted, and stored at −20 °C.

## S2. Fabrication of Aptamer-Functionalized Nylon Mesh (ApoFilter Layers)

## *S2.1 Mesh Preparation and Activation*

## Nylon mesh sheets (Lixin Huarun Mesh Co., China) were laser-cut to 11 mm diameter (BEAMO, MIRTECH Korea).

## Meshes were placed in sterile Petri dishes and incubated in 5 mL of 0.1 N HCl for 30 min.

## After acid activation, meshes were washed 3× with DI water.

## *S2.2 Surface Aldehyde Functionalization*

## Meshes were incubated in 10 mL of 2.5% glutaraldehyde (GA) (Sigma-Aldrich) for 30 min, shaking at 150 rpm.

## Following functionalization, meshes were washed 3× with DI water.

## *S2.3 Aptamer Conjugation*

For each batch:

- 50 mg of mesh was incubated with:
- 1.4 μg aptamer,
- 0.1 M EDC (1-ethyl-3-(3-dimethylaminopropyl)carbodiimide hydrochloride),
- 0.1 M NHS,
- in 5 mL of MES buffer (pH 5.5).
- Mixtures were rotated on a roller mixer for 6 h at RT.
- Excess reagents were removed by washing meshes 3× with DI water.
- Meshes were stored in PBS at 4 °C and used within 7 days.

## *S2.4 Assembly of Stacked ApoFilter Structures*

## Individual aptamer-coated meshes were stacked (ApoB layer above ApoA1 layer) using ring-shaped spacers (~300 μm thickness).

## Standard configuration = 30 layers, unless otherwise stated.

## S3. Sample Preparation

Plasma was centrifuged at 3000 × g (15 min) and filtered through 0.8 μm mesh. Pure lipoproteins were processed identically. Samples were stored at −80°C.

*S3.1 Human Plasma Handling*

- Human plasma was purchased from **Zen-Bio Inc. (USA)**.
- All samples were clarified by:
  - **3,000 × g, 15 min**
  - Filtration through **800-nm mesh**
- Aliquots were stored at **−80 °C**.

*S3.2 Preparation of Pure Lipoproteins*

- Purified **(V)LDL (#L8292)** and **HDL (#L1567)** (Merck KGaA) were diluted according to manufacturer instructions.
- Before use, samples were centrifuged:
  - **3,000 × g, 15 min**,
  - filtered through **800-nm mesh**,
    to remove aggregates or particulates.

## S4. DNase-mediated release of captured lipoproteins

*S4.1 Preparation of DNase I Elution Buffer*• DNase I elution buffer was freshly prepared immediately before use.
• The buffer consisted of:

- - DNase I: 2.8 μL
  - 10× DNase I reaction buffer: 40 μL
  - Nuclease-free water: 360 μL

• Total volume: 402.8 μL.
• DNase I and 10× DNase I buffer were thawed on ice prior to preparation.

*S4.2 Pre-equilibration of DNase I Buffer*
• The prepared DNase I elution buffer was pre-heated at 37 °C for 15 min in a humidified incubator before application to the ApoFilter.

*S4.3 DNase I Treatment and Elution Procedure*
• For each ApoFilter module, 400 μL of DNase I elution buffer was loaded onto the aptamer-functionalized mesh.
• The module was incubated at room temperature (~21 °C) for 10 min.
• During incubation, the filter was vortexed for 30 s to enhance buffer penetration and enzymatic access to immobilized aptamers.
• This incubation–vortexing cycle was repeated three times.

*S4.4 Collection and Termination of DNase Activity*
• After DNase I treatment, the eluate containing released lipoproteins was collected.
• DNase activity was terminated by buffer exchange during subsequent handling; no additional chemical quenching agents were used.
• No further cleanup steps were performed unless required for downstream analyses.

## S5. Confocal Imaging

*S5.1 Sample Preparation*

- FAM-labeled aptamers (BioLegend) were conjugated to mesh as described.
- Lipoproteins were labeled with **Cy5** (BioLegend).

*S5.2 Imaging Conditions*

- Microscope: **Zeiss LSM800**
- Objectives:
  - **100× oil**, NA 1.4 (lipoproteins)
  - **63× oil**, NA 1.4 (cells, mesh overview)
- Line averaging: **4×**
- Z-stack spacing: **0.3 μm**

*S5.3 Image Analysis*

- Software: **ZEN Black**, **FIJI (ImageJ)**
- Colocalization: **JACoP plugin (Pearson, RWC, particle-based colocalization)**
- Dual-labeled fraction quantified by center-of-mass coincidence.

## S6. SEM and TEM Imaging

*S6.1 Sample Adsorption & Staining*

- Grids: **Formvar-coated copper grids (150 mesh)**
- 15 µL lipoprotein sample was absorbed for **1 min**.
- Negative staining with **1% uranyl acetate** applied dropwise at 90° angle.
- Dried on filter paper for **10 min**.

*S6.2 Imaging*

- TEM system: **JEM-1400 Flash (JEOL)**
- Voltage: **120 kV**

## S7. Nanoparticle Tracking Analysis (NTA)

*S7.1 Measurement Conditions*

- Instrument: **ZetaView PMX-220**
- Sample dilution: particle-per-frame = **140–200**
- Camera sensitivity: **92**
- Shutter: **70**
- Video: **11 positions × 60 frames**

*S7.2 Analysis Settings*

- ZetaView software v8.02.31
- Size range: **5–1,000 nm**
- Minimum brightness: **20**
- Completed tracks: **>1,000 per sample**

**S8. Immunofluorescent Lipoprotein Labeling**

- ApoB100-Alexa647 and ApoA1-Alexa488 (R&D Systems)
- Mixed 1:10 with capture fraction
- Incubated 24 h at 37 °C

**S9. BCA Protein Quantification**

- Pierce BCA kit (#23225, Thermo Scientific)
- Standards: 0–2,000 μg/mL BSA
- Reaction:
  - 100 μL sample + 2 mL reagent,
  - 37 °C, 30 min
- Detection: 562 nm, DS-11 spectrophotometer (Denovix)

**S10. Western Blotting**

*S10.1 Protein Preparation*

- EV/lipoprotein samples mixed with Laemmli buffer + 2-ME
- Heating: 95 °C, 10 min

*S10.2 SDS-PAGE & Transfer*

- Gel: Mini-PROTEAN TGX (Bio-Rad)
- Transfer: PVDF membranes

*S10.3 Antibodies*

- EV markers: CD9, CD63, CD81(Abcam)
- Lipoproteins: ApoA1, ApoB100 (R&D Systems)
- Secondary: Goat anti-rabbit IgG-HRP (Abcam)

S10.4 Detection

- ECL reagent
- Imaging: ChemiDoc XRS+

**S11. ELISA for EV and Lipoprotein Quantification**

*S11.1 Plate Coating*

- 5 μg/mL of anti-CD9, anti-CD63, anti-CD81, anti-ApoA1, or anti-ApoB100
- 200 μL/well, 37 °C, 2 h
- Blocking: **0.5% casein**, 1 h

*S11.2 Sample & Detection*

- **100 μL sample**, 37 °C, 2 h
- Biotinylated antibodies (1 μg/mL), **1 h**
- Streptavidin-HRP (**66 ng/mL**), **1 h**

*S11.3 Signal Development*

- TMB substrate, **15 min**
- Stop: 2 M H₂SO₄, 50 μL
- Read at 450 nm (SPECTROstar Nano)

## S12. EV Isolation Procedures

*S12.1 Ultracentrifugation (UC)*

1. Clarification:
   - 3,000 × g, 15 min → 12,000 × g, 30 min
2. UC step:
   - 120,000 × g, 2 h, 4 °C
3. Wash pellet and repeat UC
4. Final resuspension: 200 μL PBS

*S12.2 Size-Exclusion Chromatography (SEC)*

- Column: qEVoriginal 70 nm (Izon Science)
- Load: 900 μL plasma
- Collect EV fractions: F8–F10
- Pool to 1.5 mL

S12.3 ExoTFF Isolation

- Load 10 mL sample into syringe-type ExoFilter
- Oscillatory TFF operation performed manually
- Lipoproteins/proteins <30 nm removed through membrane
- EVs eluted with 10 mL 1 M NaCl
- Final recovery in 2 mL PBS

## S13. ApoFilter Filtration Protocol

- Hybrid (ApoA1 + ApoB100) meshes stacked vertically.
- Filtration performed by gravity.
- Capture fractions recovered using DNase treatment.
- Permeate collected for EV isolation.


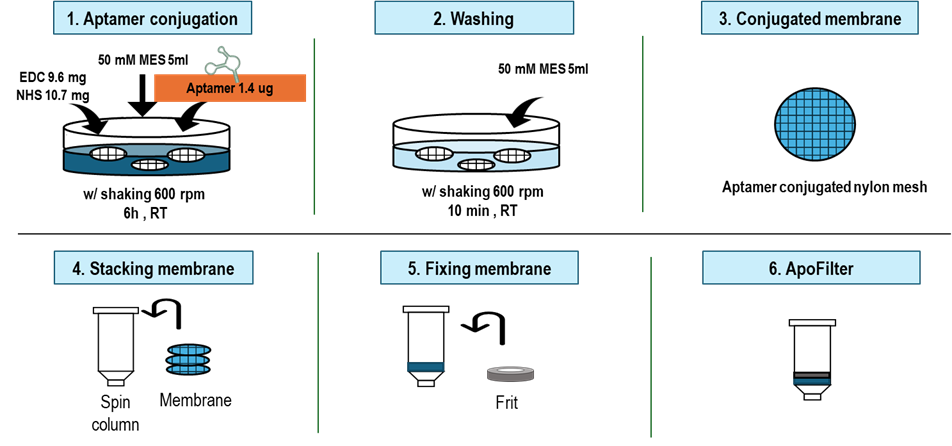
**Figure S1. Fabrication process of aptamer-conjugated nylon mesh for ApoFilter.** (1)  Covalent conjugation of aptamers using EDC/NHS chemistry in MES buffer; (2) Post-conjugation washing with MES buffer to remove unbound reagents; (3) Resulting aptamer-conjugated nylon membrane; (4–5) Stacking of membranes into a spin column housing with frit support; (6) Assembly of the final LipoFilter device for lipoprotein capture.


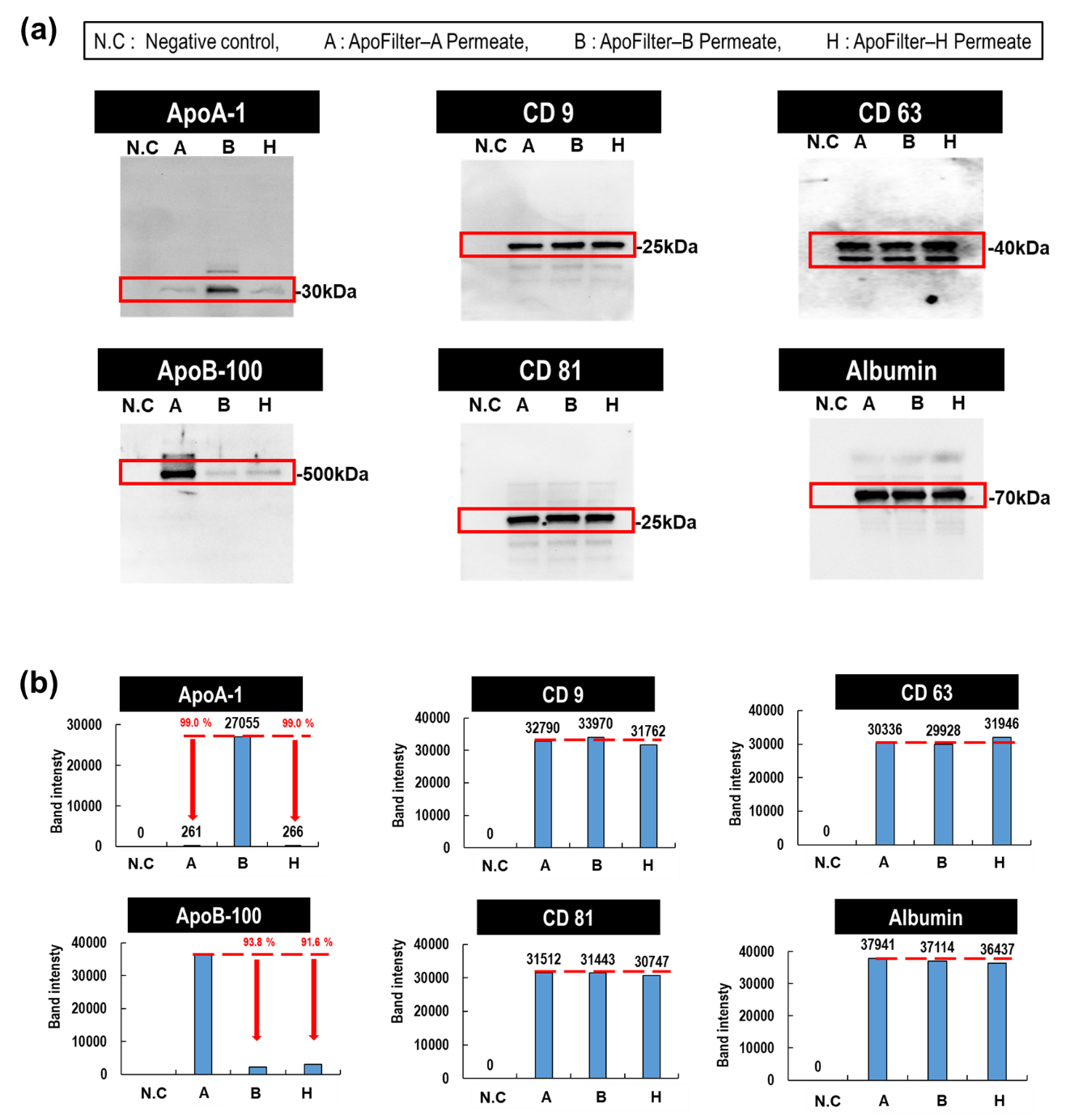


**Figure S2. Protein-level validation of ApoFilter permeate fractions by Western blot and band intensity analysis.** (a) Representative Western blot images for ApoA1, ApoB100, CD9, CD63, CD81, and albumin across negative control (N.C.), ApoFilter-A permeate (A), ApoFilter-B permeate (B), and ApoFilter-H permeate (H) samples; (b) Quantitative band intensity analysis of Western blot results shown in (a), demonstrating near-complete depletion of ApoA1 and ApoB100 and stable retention of EV markers and albumin in the processed permeate.

ApoA1 and ApoB100 bands are effectively depleted in all ApoFilter permeates, indicating selective removal of lipoproteins, while EV markers (CD9, CD63, CD81) and albumin are preserved.


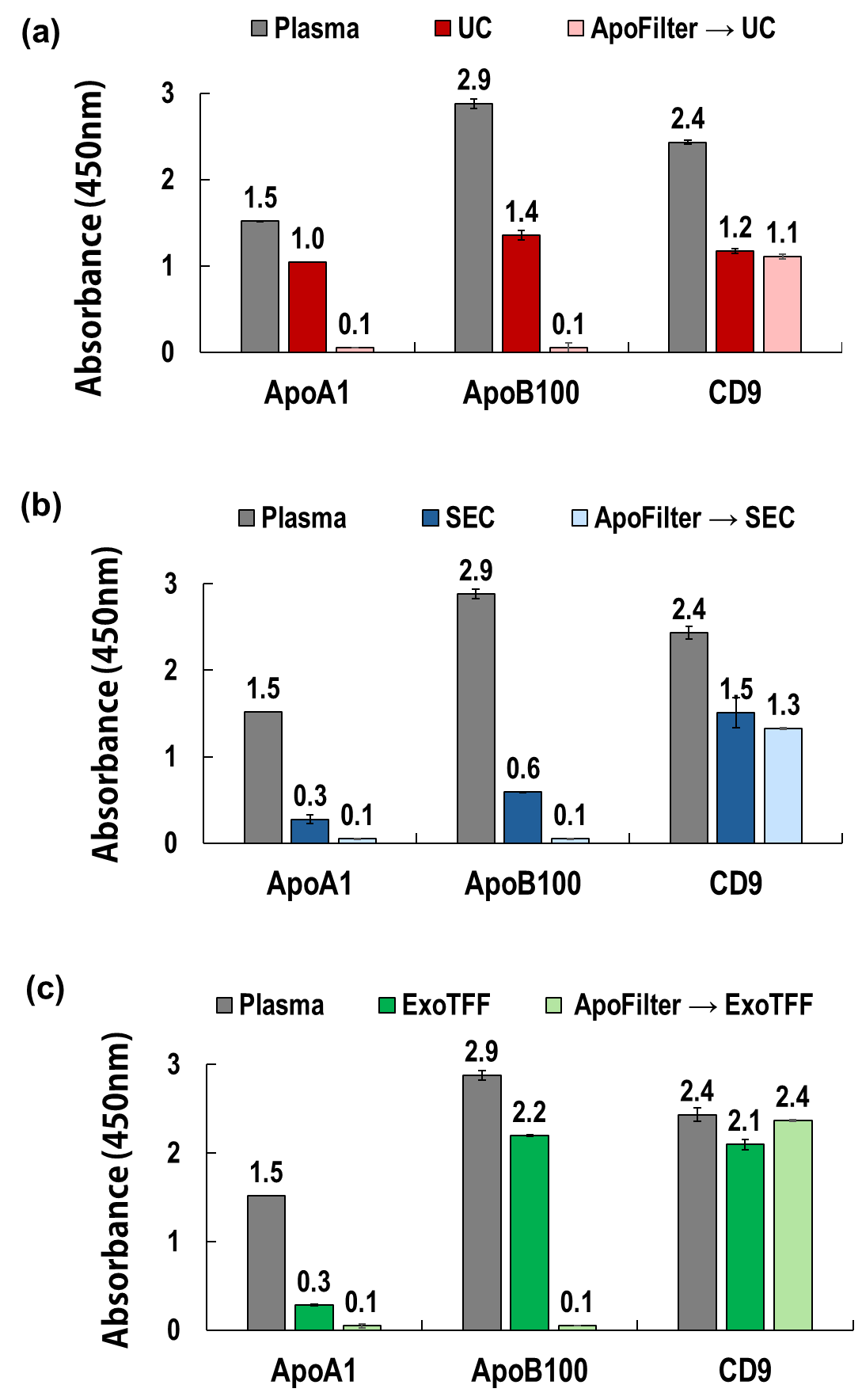


**Figure S3. ELISA quantification of ApoA1, ApoB100, and CD9 levels in plasma samples and in extracellular vesicle (EV) fractions isolated by three methods** (a) ultracentrifugation (UC), (b) size-exclusion chromatography (SEC), and (c) electrokinetic tangential flow filtration (ExoTFF)—with and without prior ApoFilter treatment. Bars indicate the absorbance at 450 nm for each marker across native plasma, conventional EV isolation workflow, and sequential application of ApoFilter followed by each isolation technique. ApoFilter pretreatment resulted in near-complete depletion of lipoprotein contaminants (ApoA1 and ApoB100), while preserving EV marker (CD9) recovery across all workflows. Numerical values above bars denote absorbance values for corresponding sample conditions.
